# Evaluation of an Ultrasound-Based Navigation System for Spine Neurosurgery: A Porcine Cadaver Study

**DOI:** 10.3389/fonc.2021.619204

**Published:** 2021-03-04

**Authors:** Houssem-Eddine Gueziri, Oded Rabau, Carlo Santaguida, D. Louis Collins

**Affiliations:** ^1^ McConnell Brain Imaging Centre, Montreal Neurological Institute and Hospital, McGill University, Montreal, QC, Canada; ^2^ Department of Neurology and Neurosurgery, McGill University, Montreal, QC, Canada

**Keywords:** ultrasound neuronavigation, spine surgery, image-guided neurosurgery, registration, evaluation, accuracy

## Abstract

**Background:**

With the growing incidence of patients receiving surgical treatment for spinal metastatic tumours, there is a need for developing cost-efficient and radiation-free alternatives for spinal interventions. In this paper, we evaluate the capabilities and limitations of an image-guided neurosurgery (IGNS) system that uses intraoperative ultrasound (iUS) imaging for guidance.

**Methods:**

Using a lumbosacral section of a porcine cadaver, we explored the impact of CT image resolution, ultrasound depth and ultrasound frequency on system accuracy, robustness and effectiveness. Preoperative CT images with an isotropic resolution of , and were acquired. During surgery, vertebrae L1 to L6 were exposed. For each vertebra, five iUS scans were acquired using two depth parameters (5 cm and 7 cm) and two frequencies (6 MHz and 12 MHz). A total of 120 acquisition trials were evaluated. Ultrasound-based registration performance is compared to the standard alignment procedure using intraoperative CT. We report target registration error (TRE) and computation time. In addition, the scans’ trajectories were analyzed to identify vertebral regions that provide the most relevant features for the alignment.

**Results:**

For all acquisitions, the median TRE ranged from 1.42 mm to 1.58 mm and the overall computation time was 9.04 s ± 1.58 s. Fourteen out of 120 iUS acquisitions (11.66%) yielded a level-to-level mismatch (and these are included in the accuracy measurements reported). No significant effect on accuracy was found with CT resolution (*F*_(2,10)_ = 1.70, *p* = 0.232), depth (*F*_(1,5)_ = 0.22, *p*= 0.659) nor frequency (*F*_(1,5)_ = 1.02, *p* = 0.359). While misalignment increases linearly with the distance from the imaged vertebra, accuracy was satisfactory for directly adjacent levels. A significant relationship was found between iUS scan coverage of laminae and articular processes, and accuracy.

**Conclusion:**

Intraoperative ultrasound can be used for spine surgery neuronavigation. We demonstrated that the IGNS system yield acceptable accuracy and high efficiency compared to the standard CT-based navigation procedure. The flexibility of the iUS acquisitions can have repercussions on the system performance, which are not fully identified. Further investigation is needed to understand the relationship between iUS acquisition and alignment performance.

## Introduction

Advancing technology and improvement in surgical techniques have contributed to the rising incidence of patients receiving surgical treatment for spinal metastatic tumours (1, 2). In the last two decades, significant efforts have been made to develop image-guided neurosurgery (IGNS) systems for spine oncology in traditional open surgery (3, 4), in minimally invasive and robotic surgeries (5–7) and in ablative therapy (8–11). Neuronavigation performed by IGNS allows the digital tracking of surgical instruments with respect to diagnostic imaging, therefore facilitating tumour localization, anatomy visualization and monitoring surgical progress. For most commercial IGNS systems, computed tomography (CT) is the preferred imaging modality for spine interventions. CT images yield good visualization of bone anatomy, suitable for fusion instrumentation. In addition, the accessibility of mobile scanners offers some flexibility for intraoperative imaging in the operating room (OR). However, there is a non-negligible risk of ionizing radiation exposure to the patient and, perhaps more impotently to the surgical staff, associated with intraoperative CT imaging (12). Alternative approaches have investigated magnetic resonance (MR) imaging to reduce radiation exposure (13–16). Although intraoperative MR provides high image resolution and good soft tissue contrast, restrictions due to high costs and ferromagnetic compatibility of surgical instruments limit its application in the OR.

Recently, the use of intraoperative ultrasound (iUS) has gained attention for spinal neuronavigation (17). While the many advantages to using iUS imaging include safety, real-time acquisition, cost-efficiency and reduced footprint in the OR, the role of iUS in spinal surgery is not fully defined and remains under investigation (18). Ultrasound acquisition has some limitations in the OR. For example, the small field of view obtained from an iUS scan reduces the ability to observe deep structures, limiting its application to mostly posterior surgical approaches. Moreover, low ultrasound signal propagation through dense bone tissues induces shadow artifacts and makes navigation using iUS images challenging. To address these limitations, iUS-based IGNS systems do not use ultrasound images for diagnosis or visual navigation. Rather, iUS is used to collect anatomical features in order to establish patient alignment with preoperative CT or MR images. Then, the navigation is performed on the preoperative images.

In our previous work (19), we introduced an open-source and freely available IGNS system based on iUS imaging which allows for CT-to-iUS image alignment for spine instrumentation. Although the system was able to achieve satisfactory results for the alignment of one vertebra at a time, the full capabilities of such a system in variable acquisition conditions are unknown. This paper investigates the limitations associated with the usability of the system on a porcine cadaver. Specifically, we are interested in the following questions: (i) is there a specific CT and/or ultrasound imaging parameters that impact the accuracy of the system? (ii) how do alignment errors, located on a specific vertebra, propagate to adjacent vertebral levels? and (iii) can we identify patterns of ultrasound acquisitions that affect accuracy? In a controlled experimental study, we explore the feasibility of ultrasound-based neuronavigation for the lumbar spine. We report the results in terms of accuracy, robustness and effectiveness and discuss the usability in clinical conditions.

## Materials and Methods

### Navigation System

**Figure 1:**
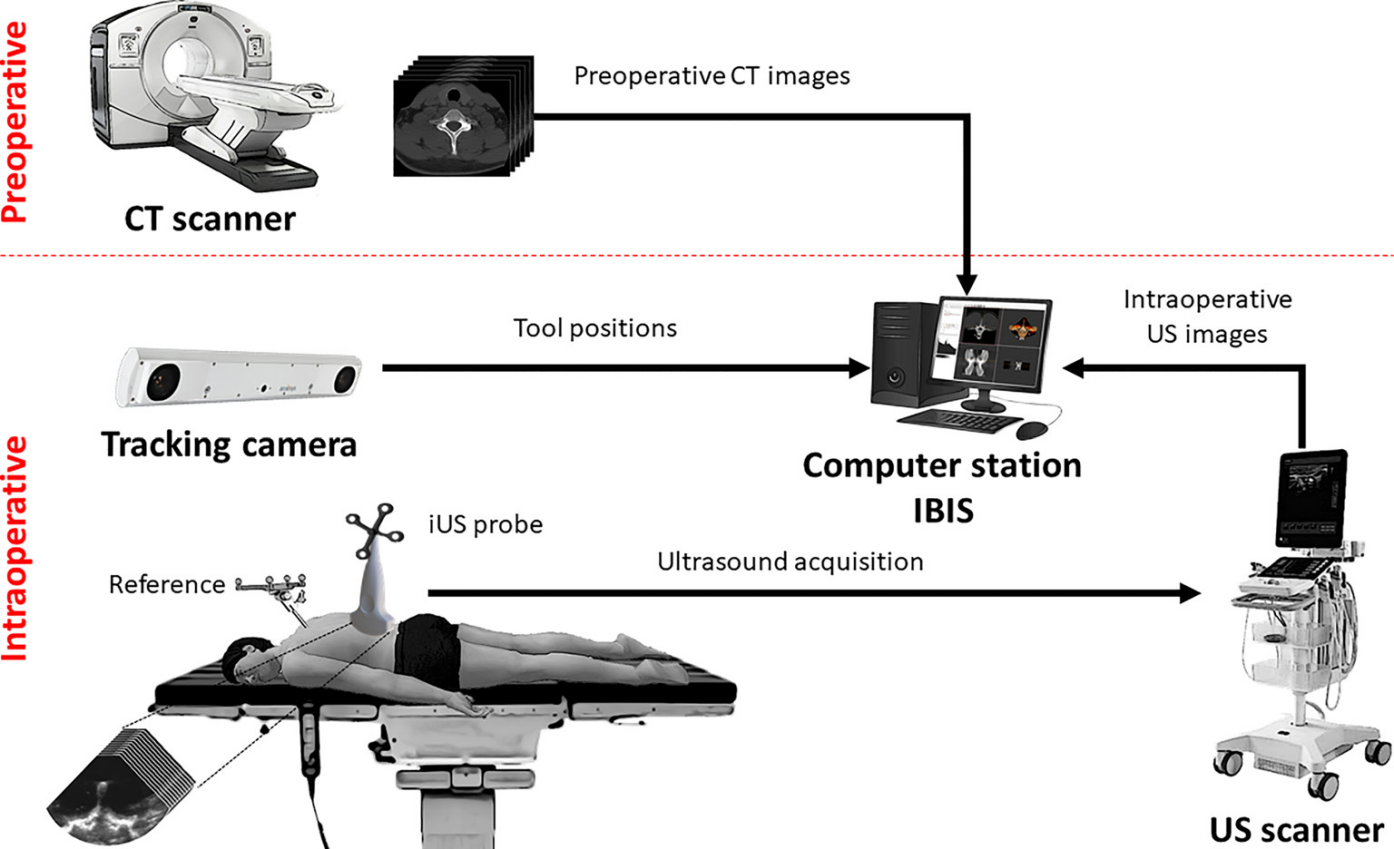
Setup of the ultrasound-based image-guided neurosurgery system.

The IGNS system is composed of three main components (**Figure 1**): an ultrasound scanner, a tracking camera and a computer station. The first component is an ultrasound unit with a linear probe (BK3500/14L3 probe, BK Medical, Peabody, MA, USA). The probe’s contact surface is 14 mm wide and small enough to fit inside the surgical cavity to acquire intraoperative images. The second component is a tracking camera (FusionTrack 500, Atracsys, Puidoux, Switzerland). It is used to determine the spatial location of infrared light-reflecting spheres rigidly fixed to surgical instruments. In this study, the tracked instruments consist of a planar blunt probe PN960-556 (Medtronic, Dublin, Ireland) used as a pointer, the iUS linear probe and a rigid body reference. The instrument positions are expressed in the reference coordinate system, therefore accounting for patient movement during navigation. The third component is a computer station that runs IBIS^1^, an open-source neuronavigation software developed in our laboratory (20). IBIS provides common navigation functionalities such as 3D data visualization, ultrasound probe calibration, ultrasound acquisitions, volume reconstruction and patient registration, and has been evaluated in the operating room for brain tumour resection (21, 22).

The iUS probe is calibrated prior to the experiments. This determines the spatial correspondence between the iUS image space (in pixel) and the patient space (in millimeters) with an accuracy ranging between 0.49 mm and 0.82 mm (23). The intraoperative procedure to establish navigation is as follows: First, the open cavity is filled with a saline solution to allow ultrasound image acquisition. The operator performs an axial iUS sweep along the caudo-cranial direction, starting from the inferior to the superior aspect of the vertebra (24). The data collected data are automatically provided to the IBIS station, and the CT-to-iUS spatial correspondence is computed by aligning the hyperechoic response produced by the bone surface on the iUS images with the precomputed posterior vertebral surface extracted from CT images (19, 25). Once the registration is completed, the tracked instruments are located on preoperative CT images for navigation.

### Data

**Figure 2:**
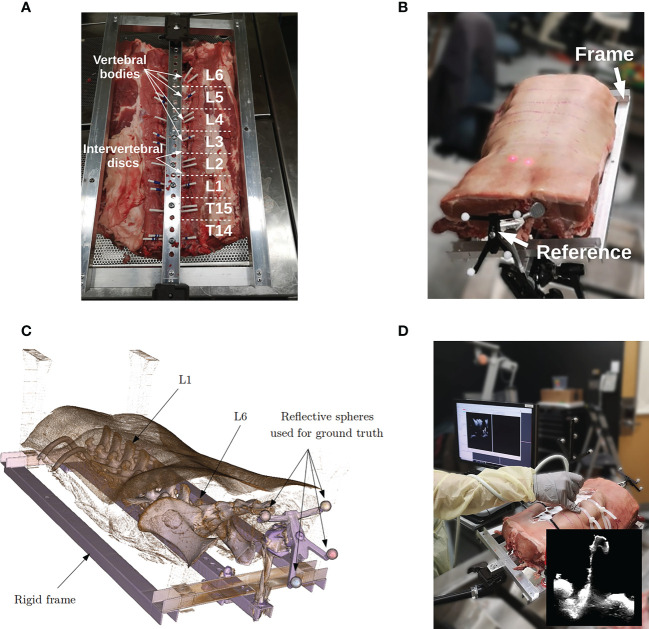
Porcine cadaver data acquisition: **(A)** specimen in supine position attached to a rigid frame, **(B)** specimen in prone position redy for surgery, **(C)** CT volume rendering of the porcine cadaver, and **(D)** intraoperative ultrasound acquisition.

Lumbar spines of porcine cadavers are commonly used for validation of spinal instrumentation due to their similarity with human specimens and the limited ethical issues they involve (26, 27). For this study, a lumbosacral section of a 80 Kg pig, in which vertebrae L1 to L6 were present, was obtained from a local butcher shop. The specimen was attached to a rigid frame to prevent intervertebral motion. A rigid body reference containing 4 infra-red reflective spheres was attached to the frame and serves as a dynamic reference object (DRO). The role of the DRO is two-fold. First, it serves as a reference coordinate space for the tracked instruments. The rigid frame ensures a fixed spatial relationship between the DRO and the specimen, allowing the instruments to be tracked with respect to the anatomy (see **Figures 2A–C**). Note that in spine surgery, the DRO is usually attached to the spinous process of the target vertebra, an adjacent vertebra or the iliac bone depending on the level of the target. Second, because the spheres are visible on the CT images, they are used to obtain a *ground truth alignment* for experiments. This procedure is similar to the one used by commercial systems that are based on intraoperative CT scanners. In this study, we compare the accuracy of our iUS-based neuronavigation system against the standard IGNS procedure based on intraoperative CT.

Before the experiment, the specimen was placed in a supine position and imaged using a clinical CT scanner (Aquilion ONE, Canon Medical, Otawara, Japan). Vertebral levels were manually identified on the CT scan. For each vertebral level, three corresponding CT volumes were reconstructed with a resolution of 0.5mm × 0.5mm × 0.5mm, 1mm × 1mm × 1mm, and 2mm × 2mm × 2mm. Then, the frame was flipped over to have the specimen in prone position and vertebrae L1 to L6 were exposed. Multiple iUS acquisitions were performed for each vertebral level with different depth and frequency parameters (see **Figure 2D**). Specifically, we investigated the effects of probe depth at 5 cm and 7 cm, and frequency of 6 and 12 MHz. The remaining ultrasound probe parameters were kept the same for all the acquisitions. For each depth–frequency combination, 5 similar independent iUS scans were acquired per vertebra yielding a total of 5 acquisitions × 2 frequencies × 2 depths × 6 vertebrae = 120 ultrasound scans. All the acquisitions were performed by the same operator. During iUS scanning, the probe was oriented in the antero-posterior direction and the 5 repetitions were performed with a slight but not significant variation in the left-right orientation and acquisition speed. This aims at reflecting the variability that may occur during a particular iUS acquisition protocol.

### Ground Truth Alignment

A single ground truth alignment is used for all the vertebrae. The alignment is obtained using a rigidly fixed DRO, similar to the procedure employed in CT-based intraoperative navigation. The procedure consists in a pair-wise matching of the position of the spheres visible on CT images with their respective position obtained from the camera tracking. The spheres appear bright on CT images and can be precisely segmented using a thresholding technique. The coordinates of each sphere’s center are then computed and used to obtain the spatial transform between the navigation space and the CT imaging space using a rigid-body landmark registration (28). This transform serves as the ground truth alignment when evaluating accuracy. Note that the validity of the ground truth alignment is subject to the assumptions that the specimen is fixed to the frame and that the spatial transform is exact. While violation of the former assumption would invalidate the ground truth, violation of the latter assumption could result in increasing ground truth misalignment as the distance from the DRO increases due to angular misalignment errors.

### Performance Metrics

The IGNS system is evaluated according to three major criteria: accuracy, robustness and effectiveness. The *accuracy* is defined as the target registration error (TRE) resulting from the difference between the alignment obtained using the proposed iUS-based method and the ground truth alignment. For each vertebral level, the TRE is given by the root mean square of the Euclidean distance computed at 7 anatomical points located on the vertebra surface, corresponding to the apex of the spinous process, left and right laminae, left and right superior articular processes and tips of the left and right transverse processes. Note that the landmarks are identified on the CT images. Therefore, the TRE gives the error between the alignment obtained with the iUS-based neuronavigation and the one obtained with standard IGNS procedure based on intraoperative CT imaging (i.e., ground truth alignment). The alignment is considered satisfactory if the TRE is below 2 mm, which is the clinical threshold suggested for spinal navigation (29). The *robustness* is measured by the success rate (in %) defined as the fraction of acquisitions that achieved satisfactory alignment, (i.e., TRE <2mm). Finally, the *effectiveness* is measured by the overall computation time required to complete the registration.

The vertebral level where the TRE is computed indicates the accuracy of the alignment at that location. This is particularly true for angular errors, where a slight angular misalignment at L6, for example, can lead to a large error at L1. Note that for commercial intraoperative CT-based IGNS systems in which the alignment is performed using the DRO’s reflective spheres, it is recommended to position the DRO near the vertebral levels receiving treatment. In our experiment, if an iUS acquisition covering a given vertebra yields a good result, we wish to identify how the accuracy propagates to other vertebral levels. Therefore, for each iUS acquisition, the TRE is computed on all vertebrae.

### Ultrasound Acquisition Coverage

**Figure 3:**
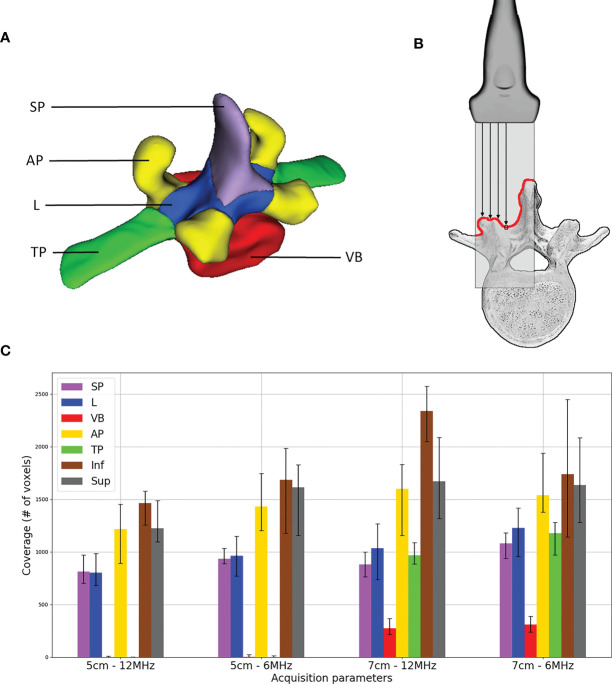
Ultrasound acquisition coverage: **(A)** labels associated with vertebra segmentation – spinous process (SP), superior and inferior articular processes (AP), laminae (L), transverse processes (TP) and pedicles and vertebral body (VB); **(B)** illustration of coverage counting, only the first voxel encountered along ultrasound image columns is considered; and **(C)** median distribution of labels’ coverage including voxels of one level inferior (Inf) and one level superior (Sup) vertebrae, error bars represent first and third quartiles.

To gain insight into how the iUS acquisition affects the alignment outcome, we examine the vertebral anatomy *coverage* produced by the ultrasound scan. In other words, the coverage is defined as how much and what part of the vertebra surface was imaged during iUS acquisition. The goal of this metric is to investigate the relationship between the iUS acquisition and the success of the alignment. To obtain the coverage, we first manually segmented the vertebrae on each CT volume at the resolution of 0.5mm × 0.5mm × 0.5mm. For each vertebra, five labels were identified (see **Figure 3A**): the spinous process (SP), laminae (L), pedicles and vertebral body (VB), inferior and superior articular processes (AP), and transverse processes (TP). In addition to these labels, we include the coverage of the vertebrae located one level inferior and one level superior. This is because the acquisition starts at the inferior most part and ends at the superior most part of the vertebra, therefore we need to account for the existing overlap between vertebrae. Then, the CT segmentation is aligned with the iUS acquisition using the ground truth transform. Finally, the coverage is given by the sum of CT voxels within the segmentation that intersect with the iUS image planes. We assume no ultrasound penetration over bone tissues, therefore, only the first voxel encountered along the iUS image column is considered in our model (see **Figure 3B**).

### Statistical Analysis

Two models were used to analyze the coverage effect on the alignment performance. The first model aims at understanding the impact that label combinations can have on the alignment outcome. Recall that the experimental design includes six vertebral levels, and for each level, two sub-groups of different frequencies and depths are constructed, each consisting of 5 repeated acquisitions. Therefore, a linear mixed-effect model is used (30). The model evaluates the TRE outcome considering every possible interaction between labels, and accounting for the group variance of vertebral levels and the sub-group variances of frequency and depth per level. The second model aims at understanding whether the coverage has an implication in causing level misalignments. The rationale is fact that coverage expresses the level location where the acquisition has been taken. In other words, if the acquisition is not centred on the correct vertebral level, we expect the coverage to be lower for the labels (SP, L, VB, AP, and TP) and higher for the adjacent levels (labels associated with inferior and superior levels). A binary response of *level misalignment* was created from the TRE results such as we associate a negative response to level misalignment if the TRE is lower than 10 mm and a positive response otherwise. Similar to the first model, we used a binomial mixed-effect model to account for group and sub-group variances of level, frequency and depth. However, because we are not interested in the different label interactions, a single independent variable representing the sum of all labels excluding adjacent levels is considered.

### Intensity Profile

Ultrasound bone appearance varies significantly depending on the acquisition and probe positioning, for example in the case of occlusions caused by shadow artifacts. In order to gain insight into how the bone surface appears in ultrasound images, we analyzed the *intensity profile* of a line passing from the bone tissue to the cavity of L5, through the lamina. The intensity profile represents the values of image intensity along a line segment. Therefore, it is possible to observe changes in intensity as the line traverses the bone surface. We analyze the intensity profile of the 20 iUS acquisitions performed on the vertebra. The median intensity and the interquartile range are reported. To compare the same segment through different acquisitions, we first align the iUS acquisitions with the CT image using the ground truth alignment. Then, the intensity profile is computed using the same line position through all acquisitions.

## Results

### Imaging Parameters

**Table 1** shows the results obtained with the different CT resolutions and ultrasound depth and frequency parameters. For all the CT volume resolutions, the median TRE ranges from 1.42 mm to 1.58 mm. Out of the 120 acquisitions, 79 (65 %) to 84 (70 %) iUS scans were successfully aligned (i.e., TRE <2mm). No significant effect was found between the accuracy and the CT resolution (*F*_(2,10)_ = 1.70, *p* = 0.232). Although a slightly lower median TRE of 1.33 mm was achieved using a depth of 5 cm and a frequency of 12 MHz, there is no significant effect of depth (*F*_(1,5)_ = 0.22, *p* = 0.659) nor frequency (*F*_(1,5)_ = 1.02, *p* = 0.359) on the accuracy. Regarding effectiveness, CT and iUS imaging parameters did not affect the computation time. The overall computation time is 9.04 s ± 1.58 s.

**Table 1:** Summary results of accuracy and computation time for different imaging parameters.

CT	US	US	Median	Successful		
Resolution	Depth	Frequency	TRE (mm)	IQR (mm)	acquisition (%)	Time (s)
0.5 mm × 0.5 mm × 0.5 mm	–	–	1.43	1.15	70.00	9.21
1 mm × 1 mm × 1 mm	–	–	1.58	1.83	65.83	8.49
2 mm × 2 mm × 2 mm	–	–	1.42	1.43	67.50	8.84
–	5 cm	6 MHz	1.47	1.76	68.89	8.33
–	5 cm	12 MHz	1.33	1.00	73.33	8.29
–	7 cm	6 MHz	1.68	1.23	64.44	9.74
–	7 cm	12 MHz	1.48	1.73	64.44	9.01

All parameters confounded are indicated by (–), TRE, target registration error; IQR, interquartile range, Successful acquisition: the fraction of iUS acquisitions achieving a TRE below 2 mm.

We observed that for some acquisitions the iUS was aligned to the wrong vertebral level, mostly one vertebral level above or below the target level. For these *vertebral level mismatch* cases, the resulting TRE is very large, typically over 10 mm. All CT resolutions confounded, such cases represent 14 out of 120 (11.67 %) acquisitions and have a median TRE of 67.17 mm ± 15.73 mm. Note that the overall TRE results presented above include those cases. If these mismatch cases are removed, the overall median TRE for all experiments is reduced, from 1.43 mm to 1.29 mm, from 1.58 mm to 1.48 mm, and from 1.42 mm to 1.32 mm for each CT resolution. Vertebral level mismatch cases are distributed as follows: 1 case at L1 (5 %), 1 case at L2 (5 %), 1 case at L3 (5 %), 2 cases at L4 (10 %), 6 cases at L5 (30 %), and 3 cases at L6 (15 %). Because of the anatomical similarity between vertebrae, it is not trivial to identify a level misalignment by visual inspection.

### Error Propagation

**Figure 4:**
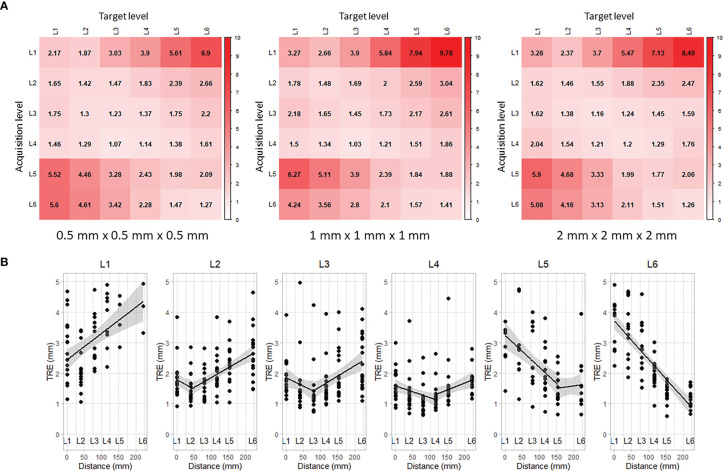
Propagation of target registration error along vertebral levels: **(A)** rows correspond to the levels where iUS acquisitions were performed and columns correspond to the levels where TRE was measured, and **(B)** linear regression of the TRE accounting for the space between vertebral levels.

The alignment results obtained for each vertebra were used to compute the TRE across all other vertebral levels. **Figure 4A** shows the propagation of the TRE for each CT resolution. Each row corresponds to the location where the iUS acquisition was performed and each column corresponds to the location where the TRE was computed. The best alignment results are located on the diagonal, i.e., close to the vertebral levels that have been imaged and used to compute the registration. Note that along the diagonal, there is no consistent increase of the TRE as the measurements are done away from the DRO located at the most inferior part of the frame (near L6). This supports the assumption regarding the validity of the ground truth alignment as the angular error is negligible. On the other hand, the error increases linearly as it moves farther from the registered vertebral level (see **Figure 4B**). The distance between each two successive spinous processes was measured to be *d*_L6/5_ = 69.36 *mm*, *d*_L5/4_ = 35.33 *mm*, *d*_L4/3_ = 38.21 *mm*, *d*_L3/2_ = 39.38 *mm*, and *d*_L2/1_ = 39.46 *mm*. For all CT resolutions, results for L1 show the worst performance with a median TRE of 3.01 *mm* ± 0.43 *mm* computed on L1, and increases to 7.96 *mm* ± 0.69 *mm* on the farthest vertebra L6 located at a distance of 221.74 mm away.

### Coverage Results

A histogram of ultrasound acquisition coverage of the labels is shown in **Figure 3C**. Note that for the additional inferior and superior levels, the entire anatomy of the vertebra was considered as a single label, therefore resulting in a higher coverage value. Among all vertebra labels, AP showed the highest coverage values (mean 1485.5 voxels) for all the scans, followed by L (mean 1004.2 voxels) and SP (mean 944.1 voxels), respectively. Labels associated with VB and TP were only visible for acquisitions with an ultrasound depth of 7 cm. VB showed the lowest coverage values as it is only visible from the gap located in the intervertebral space.

After removing the 14 outliers representing level misalignment cases, we fit the remaining TRE data with the model that includes all possible interactions between the labels. A significant relationship was found between the coverage amount of label L (*p* = 0.012) with negative correlation, as well as the combination of labels L and AP (*p* = 0.035) with positive interaction correlation, on the TRE measure. No significant effect was found for other label combinations. To identify the potential effect of coverage on causes of level misalignment, we fit the level misalignment outcomes to the binomial mixed-effect model, as described in Section 2.6. However, no statistically significant relationship was found between the acquisition coverage and level misalignment.

## Discussion

### Effect of Imaging Parameters

We conducted a thorough experiment on a porcine cadaver to investigate the capabilities and limitations of our neuronavigation system for spine surgery. The system relies on optical trackers and intraoperative ultrasound imaging to efficiently establish patient alignment, which is then used to provide neuronavigation on preoperative CT images. In this study, we evaluated the alignment quality as the resolution of preoperative CT images varies from

**Figure 5:**
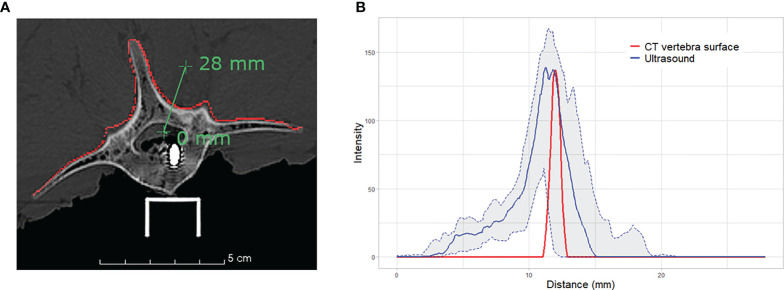
Intensity profile across the vertebral surface of L5: **(A)** illustration of the profile line (green) crossing the vertebral surface (red) at the lamina on CT image; and **(B)** plot of the intensity profiles of CT vertebral surface (red), median intensity of 20 iUS acquisitions performed on L5 (blue) and the associated interquartile range (light grey).

0.5mm × 0.5mm × 0.5mm to 2mm × 2mm × 2mm. The overall median TRE ranges between 1.42 mm and 1.58 mm across all CT resolutions, which meets the clinical accuracy requirements of 2 mm for spinal navigation (29). Although a finer CT resolution provides a better representation of anatomical details, we found no significant impact on the quality of the final alignment. This can be explained by the intrinsic characteristics related to ultrasound bone imaging, specifically, the signal variation of ultrasound response near the bone-tissue interface. The intensity at the vertebral surface varies depending on signal strength, bone density and incidence angle of the ultrasound waves caused by the probe orientation with respect to the surface. **Figure 5** illustrates the distribution of the intensity profiles crossing the lamina of L5 for all 20 ultrasound acquisitions. While the vertebral surface on the CT image can be easily extracted, even with slice thickness of 2 mm, the intensities of the ultrasound images at the vertebral surface are more widely distributed, rendering the identification of the exact location of the vertebral surface challenging.

We investigated the effects that basic ultrasound parameters, including depth and frequency, have on the alignment quality. We first explore whether anterior structures of the vertebra, located deeper in the cavity, provide relevant features for the alignment. We then tested the effect of the probe frequency on the alignment. While low probe frequencies enable deeper tissue penetration and provide information on underlying vertebral structures, high probe frequencies provide a better image quality. In our experiment, increasing ultrasound depth and frequency did not show any significant improvement in the accuracy. The iUS acquisitions are performed in open surgery, in which the posterior part of the vertebra is exposed. The ultrasounds propagate through the saline solution in the cavity with negligible signal loss before encountering the bone surface. Therefore, signal loss usually associated with tissue absorption did not play a critical role in alignment accuracy.

The IGNS system was evaluated based on the alignment of a single vertebra at a time, meaning that the information used to establish the alignment is associated with one vertebral level in the pre- and intra-operative images. This intentional design is meant to avoid errors due to intervertebral motion caused by spine curvature changes, resulting from a typical preoperative CT scan in supine position while the iUS images are acquired in prone position during surgery. Assuming no spine curvature changes, we evaluated the propagation of the alignment error along the vertebral levels. Overall, the alignment accuracy was satisfactory for directly adjacent vertebral levels, i.e., one level superior and one level inferior to the vertebra level used to establish the alignment. The accuracy is sufficient for instrumentation of three vertebral levels per iUS alignment. This reduces the number of iUS acquisitions needed to establish navigation during surgery, decreasing considerably intraoperative time and surgical workflow interruptions. However, the best accuracy results were obtained at the vertebral level where the iUS acquisition was performed. Therefore, it is recommended to perform the alignment procedure on the vertebra being instrumented. The computation time is ∼10 seconds and we estimate the iUS acquisition time to be less than one minute, which makes the alignment procedure significantly faster than the standard intraoperative CT navigation, estimated to be 15–20 min. In addition, for surgeries involving several vertebral levels, the DRO needs to be re-positioned near the instrumented vertebral level. In such a case, the intraoperative CT procedure needs to be performed again.

### Intraoperative Acquisition

The iUS acquisition plays a crucial role in the resulting quality of the alignment. In our IGNS system, the iUS acquisition is expected to be a linear scan from inferior to superior parts of the vertebra. This predefined constraint is used to initialize the alignment so that the iUS and the CT volumes are located in the same space and in the same orientation. We draw the reader’s attention to the low accuracy results obtained with L1 (see **Figure 4**). This specific limitation has been caused by difficulties encountered while performing iUS acquisitions at L1. Because we could not extend the surgical opening beyond L1 without risking the perforation of the cavity, the width of the opening at that location was narrow, restricting the probe’s motion. Note that this scenario is unlikely to happen during real surgery, as the vertebrae receiving treatment are more widely exposed. Nevertheless, this highlights an underlying limitation of iUS-based IGNS systems, in which the ultrasound probe physical dimensions need to be accounted for during data acquisition in open spine surgery. Probes with small size are to be preferred.

To understand which part of the vertebra provides the most relevant features for the alignment, we identified 5 regional vertebral labels and analyzed coverage data produced by the 120 iUS acquisitions performed during the experiment. Results revealed a significant relationship between the accuracy and the iUS coverage of laminae and superior and inferior articular processes. The negative correlation between the laminae coverage and the alignment error indicates that accuracy increases (error is reduced) with more laminae coverage. Similar results apply to the combination of laminae and articular processes coverage as a positive interaction was found between the two labels. This seems to be consistent with the fact that articular processes and laminae are the most prominent structures in posterior approaches for open spine surgery. Note that the coverage of the spinous process did not show a significant effect on accuracy. One would expect the spinous process to play a role in the alignment since it is the most exposed part of the vertebra. However, the shape of the spinous process forming a crest in the anterio-posterior direction does not represent a good anatomical feature for the iUS acquisition. This is because most of the spinous process anatomy is in the same direction as the ultrasound beams produced by a posterior scan, resulting in low signal response along the spinous process. Note that one side of the spinous process can be imaged with the probe slightly tilted to the left or to the right of the vertebra.

Although we identified vertebral regions that provide relevant features, we have been unsuccessful to specifically determine the cause of alignment failures. From the ultrasound acquisitions, we found no particular pattern that seems to increase or decrease the alignment accuracy. To reduce scan variability, and perhaps increase robustness, a combination of multiple iUS acquisitions that allows the formation of a homogeneous ultrasound volume can be explored in the future.

### Limitations

Regarding iUS acquisition, we used a linear ultrasound probe to collect intraoperative data. While linear probes have typically a higher frequency and thus a better depth image quality than curved probes, the resulting acquisitions have a smaller field of view, especially for imaging lateral parts of the vertebra. This limits the coverage of the transverse processes. In our experiment, only the proximal parts of the transverse processes were visible when the probe’s depth was set to 7 cm. Future work will involve investigating the use of a curved probe for spinal IGNS, which would provide a wider field of view.

The model presented to quantify the ultrasound acquisition coverage has two major limitations. First, the model assumes no bone penetration, meaning that the coverage only accounts for the first voxel at the surface of the vertebra. While this allows identifying parts of the vertebra prominently exposed to the ultrasound, it does not account for underlying tissues that may result be imaged, especially for low frequency acquisitions. The second limitation involves the oversimplification of the ultrasound wave propagation. In our model, we assume the propagation to be linear along the ultrasound image columns, therefore, only accounting for the longitudinal ultrasound wave travel. In reality, the propagation has a transverse wave component that allows objects adjacent to the ultrasound image column to be imaged. Moreover, the oversimplification of the ultrasound wave does not consider the angular incidence of the beam on the bone surface. As a result, the model does not account for the intensity response of the ultrasound images. A more complex model would provide more accurate coverage information as it would include reflections and bone penetration which characterize the visibility of the vertebral surface on iUS images.

Finally, measurement of accuracy was carried out under the assumption of a rigid spine. The vertebrae were fixed to a metal frame to prevent intervertebral motion. Therefore, the DRO could be placed at a distant location, i.e., the edge of the frame, without invalidating the TRE measurements. In surgery, the spine curvature is subject to variation due to instrumentation, respiratory motion and patient positioning. The placement of the DRO for neuronavigation is critical as a distant DRO could result in large inaccuracies (31). Further work is needed to investigate how spine curvature changes may affect accuracy.

## Conclusion

In this paper, an ultrasound-based neuronavigation system for spine surgery was evaluated on a porcine cadaver. The overall accuracy of the system ranges between 1.42 mm and 1.58 mm meeting the clinical accuracy requirement of 2 mm for spine navigation. The system demonstrated high robustness to different CT resolutions, ultrasound depth and frequency parameters. We analyzed multiple ultrasound acquisitions and identified both laminae and articular processes to provide relevant features for image alignment. However, the system suffers some limitations related to the intraoperative acquisitions with ultrasound. Fourteen out of the 120 trials resulted in a one-level-off misalignment. These misalignments can be difficult to identify visually and require particular attention. Additional work needs to be carried out to understand ultrasound acquisition patterns that yield the best alignment results.

## Data Availability Statement

The raw data supporting the conclusions of this article will be made available by the authors, without undue reservation.

## Ethics Statement

The study involves a section of pig purchased from a local certified butcher. Ethical review and approval were not required for this study.

## Author Contributions

H-EG, CS, and LC contributed to conception and design of the study. H-EG and OR conducted experimentation. H-EG and LC contributed to data analysis. H-EG contributed to software development and wrote original manuscript. All authors contributed to the article and approved the submitted version.

## Funding

This study was funded by grants from the Canadian Institutes of Health Research (No. 246067) and the Natural Sciences and Engineering Research Council of Canada (No. 396395).

## Conflict of Interest

The authors declare that the research was conducted in the absence of any commercial or financial relationships that could be construed as a potential conflict of interest.

## Footnotes

1 Intra-operative Brain Imaging System: http://ibisneuronav.org/

## References

[1] Yoshihara H Yoneoka D. Trends in the surgical treatment for spinal metastasis and the in-hospital patient outcomes in the United States from 2000 to 2009. Spine J (2014) 14:1844–9. 10.1016/j.spinee.2013.11.029 24291034

[2] Fehlings MG Nater A Holmer H. Cost-effectiveness of surgery in the management of metastatic epidural spinal cord compression: a systematic review. Spine (2014) 39:S99–S105. 10.1097/BRS.0000000000000525 25077913

[3] Nasser R Drazin D Nakhla J Al-Khouja L Brien E Baron EM. Resection of spinal column tumors utilizing image-guided navigation: a multicenter analysis. Neurosurgical Focus FOC (2016) 41:E15. 10.3171/2016.5.FOCUS16136 27476839

[4] Ando K Kobayashi K Machino M Ota K Morozumi M Tanaka S. Computed tomography-based navigation system-assisted surgery for primary spine tumor. J Clin Neurosci (2019) 63:22–6. 10.1016/j.jocn.2019.02.015 30827883

[5] Nagashima H Nishi T Yamane K Tanida A. Case report: osteoid osteoma of the c2 pedicle: surgical technique using a navigation system. Clin Orthopaedics Related Research® (2010) 468:283. 10.1007/s11999-009-0958-8 PMC2795842 19568822

[6] Campos WK Gasbarrini A Boriani S. Case report: curetting osteoid osteoma of the spine using combined video-assisted thoracoscopic surgery and navigation. Clin Orthopaedics Related Research® (2013) 471:680–5. 10.1007/s11999-012-2725-5 PMC3549152 23212772

[7] Sayari AJ Pardo C Basques BA Colman MW. Review of robotic-assisted surgery: what the future looks like through a spine oncology lens. Ann Trans Med (2019) 7:224–4. 10.21037/atm.2019.04.69 PMC6595200 31297389

[8] Tatsui CE Lee SH Amini B Rao G Suki D Oro M. Spinal Laser Interstitial Thermal Therapy: A Novel Alternative to Surgery for Metastatic Epidural Spinal Cord Compression. Neurosurgery (2016) 79:S73–82. 10.1227/NEU.0000000000001444 27861327

[9] Morassi LG Kokkinis K Evangelopoulos DS Karargyris O Vlachou I Kalokairinou K. Percutaneous radiofrequency ablation of spinal osteoid osteoma under ct guidance. Br J Radiol (2014) 87:20140003. 10.1259/bjr.20140003 24712322 PMC4075560

[10] Wallace AN Greenwood TJ Jennings JW. Use of imaging in the management of metastatic spine disease with percutaneous ablation and vertebral augmentation. Am J Roentgenol (2015) 205:434–41. 10.2214/AJR.14.14199 26204297

[11] Yu F Niu XH Zhang Q Zhao HT Xu LH Deng ZP. Radiofrequency ablation under 3d intraoperative iso-c c-arm navigation for the treatment of osteoid osteomas. Br J Radiol (2015) 88:20140535. 10.1259/bjr.20140535 26415989 PMC4984927

[12] Mendelsohn D Strelzow J Dea N Ford NL Batke J Pennington A. Patient and surgeon radiation exposure during spinal instrumentation using intraoperative computed tomography-based navigation. Spine J (2016) 16:343–54. 10.1016/j.spinee.2015.11.020 26686604

[13] Woodard EJ Leon SP Moriarty TM Quinones A Zamani AA Jolesz FA. Initial experience with intraoperative magnetic resonance imaging in spine surgery. Spine (2001) 26:410–7. 10.1097/00007632-200102150-00018 11224889

[14] Takahashi S MoriKawa S Saruhashi Y MatsUsue Y Kawakami M. Percutaneous transthoracic fenestration of an intramedullary neurenteric cyst in the thoracic spine with intraoperative magnetic resonance image navigation and thoracoscopy. J Neurosurg: Spine (2008) 9:488–92. 10.3171/SPI.2008.9.11.488 18976180

[15] Takahashi S Saruhashi Y Odate S Matsusue Y Morikawa S. Percutaneous aspiration of spinal terminal ventricle cysts using real-time magnetic resonance imaging and navigation. Spine (2009) 34:629–34. 10.1097/BRS.0b013e31819b33d6 19282744

[16] Tatsui CE Nascimento CNG Suki D Amini B Li J Ghia AJ. Image guidance based on mri for spinal interstitial laser thermotherapy: technical aspects and accuracy. J Neurosurg: Spine (2017) 26:605–12. 10.3171/2016.9.SPINE16475

[17] Gueziri HE Santaguida C Collins DL. The state-of-the-art in ultrasound-guided spine interventions. Med Image Anal (2020) 65:101769. 10.1016/j.media.2020.101769 32668375

[18] Prada F Vetrano IG Filippini A Del Bene M Perin A Casali C. Intraoperative ultrasound in spinal tumor surgery. J Ultrasound (2014) 17:195–202. 10.1007/s40477-014-0102-9 25177392 PMC4142127

[19] Gueziri HE Yan CX Collins DL. Open-source software for ultrasound-based guidance in spinal fusion surgery. Ultrasound Med Biol (2020) 46(12):3353–68. 10.1016/j.ultrasmedbio.2020.08.005 32907772

[20] Drouin S Kochanowska A Kersten-Oertel M Gerard IJ Zelmann R De Nigris D. IBIS: an OR ready open-source platform for image-guided neurosurgery. Int J Comput Assisted Radiol Surg (2017) 12:363–78. 10.1007/s11548-016-1478-0 27581336

[21] Mercier L Araujo D Haegelen C Del Maestro RF Petrecca K Collins DL. Registering pre- and postresection 3-dimensional ultrasound for improved visualization of residual brain tumor. Ultrasound Med Biol (2013) 39:16–29. 10.1016/j.ultrasmedbio.2012.08.004 23200177

[22] Gerard IJ Kersten-Oertel M Drouin S MD JAH Petrecca K Nigris DD. Combining intraoperative ultrasound brain shift correction and augmented reality visualizations: a pilot study of eight cases. J Med Imaging (2018) 5:1–12. 10.1117/1.JMI.5.2.021210 PMC5786643 29392162

[23] Mercier L Del Maestro RF Petrecca K Kochanowska A Drouin S Yan CX. New prototype neuronavigation system based on preoperative imaging and intraoperative freehand ultrasound: system description and validation. Int J Comput Assisted Radiol Surg (2011) 6:507–22. 10.1007/s11548-010-0535-3 20886304

[24] Gueziri HE Collins DL. Fast registration of ct with intra-operative ultrasound images for spine surgery. In: Computational Methods and Clinical Applications for Spine Imaging. Cham: Springer International Publishing (2018). p. 29–40. 10.1007/978-3-030-13736-6_3

[25] Gueziri HE Drouin S Yan CXB Collins DL. Toward real-time rigid registration of intra-operative ultrasound with preoperative CT images for lumbar spinal fusion surgery. Int J Comput Assisted Radiol Surg (2019) 14:1933–43. 10.1007/s11548-019-02020-1 31254179

[26] Dath R Ebinesan A Porter K Miles A. Anatomical measurements of porcine lumbar vertebrae. Clin Biomech (Bristol Avon) (2007) 22:607–13. 10.1016/j.clinbiomech.2007.01.014 17360085

[27] Sheng SR Wang XY Xu HZ Zhu GQ Zhou YF. Anatomy of large animal spines and its comparison to the human spine: a systematic review. Eur Spine J (2010) 19:46–56. 10.1007/s00586-009-1192-5 19876658 PMC2899734

[28] Schroeder W Martin K Lorensen B. The Visualization Toolkit An Object-Oriented Approach To 3D Graphics Fourth Edition. New York, United States: Kitware (2006). 10.1016/B978-012387582-2/50003-4

[29] Cleary K Anderson J Brazaitis M Devey G DiGioia A Freedman M. Final report of the technical requirements for image-guided spine procedures workshop. Comput Assisted Surg (2000) 5:180–215. 10.1002/1097-0150(2000)5:3<180::AID-IGS6>3.0.CO;2-C 10964090

[30] Quené H van den Bergh H. Examples of mixed-effects modeling with crossed random effects and with binomial data. J Memory Lang (2008) 59:413–25. 10.1016/j.jml.2008.02.002

[31] Quiñones-Hinojosa A Kolen ER Jun P Rosenberg WS Weinstein PR. Accuracy over space and time of computer-assisted fluoroscopic navigation in the lumbar spine in vivo. Clin Spine Surg (2006) 19:109–13. 10.1097/01.bsd.0000168513.68975.8a 16760784

